# One-week test-retest recordings of resting cardiorespiratory data for reliability analysis

**DOI:** 10.1038/s41597-024-04303-y

**Published:** 2025-01-03

**Authors:** Andy Schumann, Franziska Lukas, Katrin Rieger, Yubraj Gupta, Karl-Jürgen Bär

**Affiliations:** Lab for Autonomic Neuroscience, Imaging and Cognition (LANIC), Department of Psychosomatic Medicine and Psychotherapy, Jena University Hospital, Jena, Germany

**Keywords:** Cardiovascular biology, Predictive markers

## Abstract

Heart rate variability (HRV) is a key indicator of cardiac autonomic function, making reliable assessment crucial. To examine the test-retest stability of resting HRV in healthy individuals, fifty participants attended two lab sessions within a week, at the same time of day. After a 5-minute acclimatization period, electrocardiogram and respiration were recorded at rest. For validation, average heart rate and RMSSD were assessed over 15 minutes using a validated open-source toolbox. Test-retest agreement was evaluated using intra-class correlation (ICC), and coefficients of variation (CV). Mean heart rate showed high stability (ICC = 0.81, CV = 6%), while RMSSD had lower concordance (ICC = 0.75) and greater variation (CV = 30%). These findings indicate good test-retest agreement for standard HRV features. However, a wide range of methodologies exists for assessing various properties of heart rate dynamics. This database is intended to support other researchers in testing additional HRV metrics to evaluate their reliability in healthy individuals.

## Background & Summary

Over the past few decades, heart rate variability (HRV) has become a reliable predictor of cardiovascular risk and overall mortality^1^. HRV measures fluctuations in heartbeat intervals influenced by both sympathetic and parasympathetic nervous systems. Low HRV has been linked to various medical conditions, including hypertension, chronic kidney disease, stroke, and dementia^2–7^. Given its clinical importance, accurately assessing HRV is crucial.

A review by Sandercock *et al*.^8^ found significant variability in HRV test-retest reliability, influenced by the study population, the type of HRV metric, and the test-retest interval^8^. HRV is commonly measured using time-domain (e.g., standard deviation of heartbeat intervals) and frequency-domain analyses (e.g., low- and high-frequency components)^9^. Nonlinear approaches have also been proposed for a more robust assessment of heart rate dynamics^10–12^. The reliability of HRV depends on the recording duration, with short-term (5–15 minutes) and long-term (24 hours) measurements providing different insights^8^.

At rest, there is a significant effect of respiration on HRV, a phenomenon known as respiratory sinus arrhythmia (RSA), where heart rate increases during inhalation and decreases during exhalation^13–15^. This cardiorespiratory coupling reflects the dynamic interaction between the autonomic nervous system and cardiac function, primarily mediated by vagal activity^16^. Different methods have been developed to assess this coupling. One common approach is time-domain analysis, which examines the variation in heartbeat intervals in relation to the respiratory cycle^17,18^. Frequency-domain analysis, on the other hand, measures the power of heart rate fluctuations within specific frequency bands associated with respiration, typically the high-frequency band^19^. A variety of other methods exist including nonlinear approaches such symbolic dynamics or phase-rectified signal averaging that offer a more complex assessment by evaluating the synchrony and complexity of the interactions between heart rate and respiration^20–22^.

In this study, we collected data to investigate the test-retest stability of resting HRV and cardiorespiratory coupling in healthy subjects.

## Methods

### Participants

Resting-state physiological recordings were obtained from 51 healthy volunteers (35 women, 16 men, 38 ± 16 years of age). All participants were recruited from the local community through word of mouth and advertisements on our website. One participant withdrew after the first session for personal reasons, leaving a total of 50 participants who completed both sessions in our laboratory. Demographic information is presented in Table 1.

**Table 1:** Demographic information on sample of healthy volunteers who have completed two sessions.

Feature	Mean ± SD	Range
*Age [years]*	37.7 ± 16.1	19–73
*Gender [f/m]*	n = 35 / n = 16	—
*BMI [kg/m²]*	22.9 ± 3.3	17–31

BMI: body mass index. SD: standard deviation.

### Human subjects

The study concept, including consent for pseudonymized data sharing, was approved by the ethics committee of the medical faculty of the Friedrich-Schiller-Universität Jena (#5423-02/18). All research was performed in accordance with relevant guidelines and regulations. Informed written consent were obtained from all participants including consent to publish obtained data without identifying any individuals.

### Procedures

All measurements were recorded at the Department of Psychosomatic Medicine and Psychotherapy at Jena University Hospital.

We performed two assessments one week apart at a similar time of day (mean difference 5 ± 43 min). The resting state recordings were made in the supine position. Participants were not allowed to exercise excessively, smoke or eat heavy meals in the two hours before the session.

### ECG

An ECG (lead II) was recorded at 1000 Hz by an MP150 (ECG100C, BIOPAC systems inc., Golata, CA, USA). Pre-gelled Ag/AgCl electrodes (BlueSensor VL, Ambu GmbH, Bad Nauheim, GER) on the chest according to an adjusted Einthoven triangle^23^.

### Respiration

**Fig. 1:**
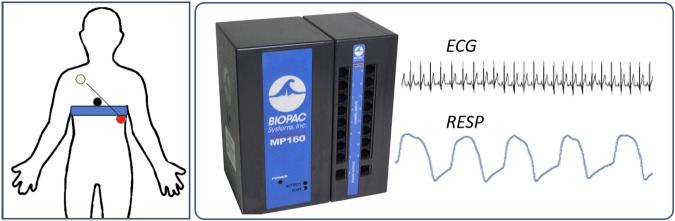
Cardiorespiratory recordings using the MP150 acquisition unit (BIOPAC Systems Inc.). Left: One ECG channel has been recorded between the electrodes red and white (lead II). A belt with a strain-gauge transducer acquired movement of the thorax during breathing. Right: The ECG and respiratory movement were sampled synchronously at 1000 Hz. Pictures from www.biopac.com adapted with permission.

Respiratory movements were recorded using a strain gauge transducer integrated into a belt, which was secured around the chest at the level of the lower end of the sternum (see Fig. 1). The acquired signal was low-pass filtered at 10 Hz and sampled at 1000 Hz (RESP100C, BIOPAC systems inc., Golata, CA, USA).

### Measurement protocol

Measurements were conducted in our temperature-controlled autonomic laboratory, maintained at 22 °C. The recording environment was quiet and fully shaded, with constant illumination provided by an indirect light source. Each session began with an interview, followed by a detailed explanation of the study’s purpose and design. Written consent was obtained from all participants.

Participants were then positioned comfortably on the examination tilt table, and electrodes and respiration belts were applied. For the resting state recording, participants were instructed to remain still and avoid movement, yawning, or coughing. A grey ellipse was displayed on a screen in front of them to allow free eye movement. An eye tracker monitored pupil size to ensure participants did not fall asleep.

Before data acquisition began, the instructor checked the signal quality and adjusted the electrodes and respiration belts if needed. Once the signal quality was deemed satisfactory, the recording proceeded for 20 minutes. The first five minutes were excluded from the analysis and removed from the data.

### Parameters

To demonstrate plausibility of collected data, we investigated test-retest stability of standard time domain heart rate variability indices. Therefore, heart beats were extracted and analyzed using the open source PhysioNet Cardiovascular Signal Toolbox implemented in MATLAB (R2019a, The Mathworks, Natick, MA, USA). This toolbox has been validated previously^24^ and is freely available at PhysioNet.org^25^.

## Data Records

Data are available at www.figshare.com (10.6084/m9.figshare.26359453.v1)^26^. Each recording is stored as a data file containing ECG and respiration signals for 15 minutes. The data are organized into columns, with the first column representing the time index in seconds, the second column containing the ECG signal in microvolts, and the third column representing the respiratory signal in volts. Additional information can be found in the table *subjects.csv* that is included in the repository.

### Data anonymization

Files were pseudonymized to numbers 01-51 after random ordering. The table *subjects.csv* gives age in years, sex (man/woman), and body mass index in kg/m² for each participant.

## Technical Validation

**Fig. 2:**
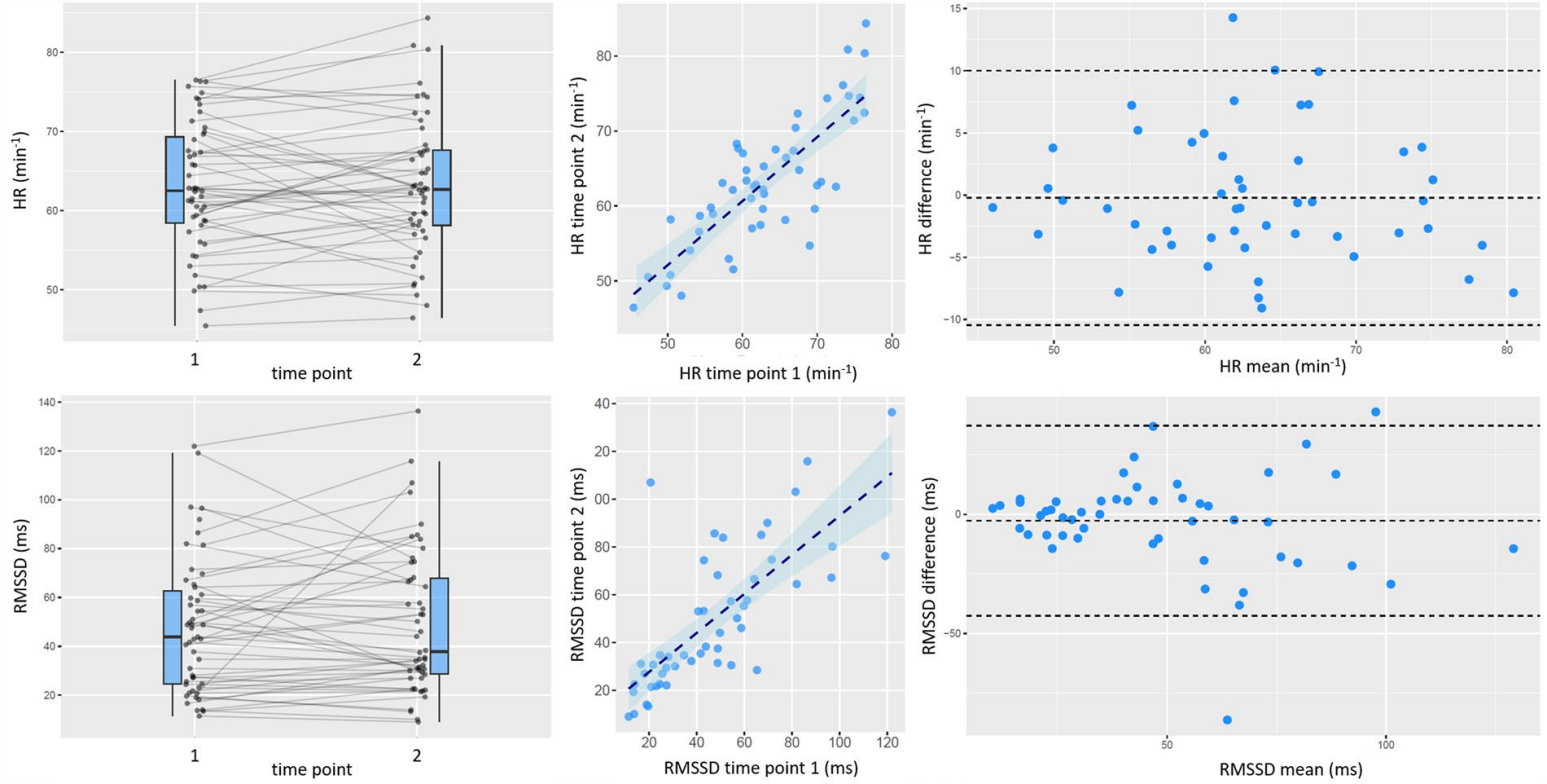
Test-retest stability of mean heart rate (HR, top row) and short-term heart rate variability (RMSSD, bottom row). Left: Changes from first to second session. Middle: Test-retest correlation. Right: Bland-Altman plots depicting differences (session 1 – session 2) over then average of both sessions. Dashed lines indicate mean difference and limits of agreement (1.96 standard deviations above and below).

To assess the validity of the recorded data, we estimated the mean heart rate (HR) and RMSSD from the entire heart beat interval time series recorded during both the test and retest sessions (see Fig. 2). Stability and concordance were evaluated using coefficients of variation (CV), calculated as the ratio of the standard deviation to the mean of the two measurements, and intraclass correlation coefficients (ICCs) (two-way mixed model, single measure, absolute agreement; Shrout and Fleiss, 1979).

As shown on the left side of Fig. 2, there were no significant differences between the test and retest estimates for either parameter. The coefficient of variation was CV = 5.8% for HR and CV = 29.6% for RMSSD. Both HR (ICC = 0.81, [0.71; 0.88]) and RMSSD (ICC = 0.75, [0.63; 0.84]) showed very high correlation between the test and retest sessions (Fig. 2, middle).

The Bland-Altman plot (Fig. 2, right side) depicts the difference between test and retest measurements over the average of the two measurements for the same subject. For HR, the mean bias was −0.2 min^−1^ with limits of agreement ranging from −10.5 to 10.0 min^−1^. The mean bias of RMSSD was −2.7 ms with limits of agreement from −42.6 to 37.2 ms. One outlier appeared to skew these statistics; specifically, in one subject, a high prevalence of premature heartbeats significantly elevated the RMSSD value during the second session (‘51_2.txt’).

However, even without sophisticated artifacts management or outlier correction these results indicate good reproducibility of standard HRV features that are comparable to previous reports.

## Data Availability

For the technical validation, we used code that is publicly available without restrictions (see methods section). In detail following functions/scripts were used: *jqrs.m* - QRS detector based on the Pan-Tompkins method (www.physionet.org/content/pcst) *ada_f.m* – adaptive filtering of heart beat time series (https://tocsy.pik-potsdam.de/ada.php).
